# Ovatodiolide suppresses colon tumorigenesis and prevents polarization of M2 tumor-associated macrophages through YAP oncogenic pathways

**DOI:** 10.1186/s13045-017-0421-3

**Published:** 2017-02-28

**Authors:** Yan-Jiun Huang, Ching-Kuo Yang, Po-Li Wei, Thanh-Tuan Huynh, Jacqueline Whang-Peng, Tzu-Ching Meng, Michael Hsiao, Yew-Ming Tzeng, Alexander TH Wu, Yun Yen

**Affiliations:** 1 The PhD Program for Translational Medicine, College of Medical Science and Technology, Taipei Medical University, Taipei, Taiwan; 2 Division of Colorectal Surgery, Department of Surgery, Taipei Medical University Hospital, Taipei Medical University, Taipei, Taiwan; 3 Department of Surgery, College of Medicine, Taipei Medical University, Taipei, Taiwan, People’s Republic of China; 4 Division of Colorectal Surgery, Department of Surgery, Mackay Memorial Hospital, Taipei, Taiwan; 5 Center for Molecular Biomedicine, University of Medicine and Pharmacy, Ho Chi Minh City, Vietnam; 6 Division of Cancer Center, Wan Fang Hospital, Taipei Medical University, Taipei, Taiwan; 7 Institute of Biological Chemistry, Academia Sinica, Taipei, Taiwan; 8 Genomics Research Center, Academia Sinica, Taipei, Taiwan; 9 Center for General Education, National Taitung University, Taitung, 95092 Taiwan; 10 The PhD Program of Cancer Biology and Drug Discovery, College of Medical Science and Technology, Taipei Medical University, Taipei, Taiwan; 11 Center of Excellence for Cancer Research, Taipei Medical University, Taipei, Taiwan; 12 Department of Life Science, National Taitung University, Taitung, Taiwan

**Keywords:** M2 tumor-associated macrophages, Colon tumorigenesis, Stemness, YAP1, Ovatodiolide

## Abstract

**Background:**

An increased expression of Yes-associated protein (YAP1) has been shown to promote tumorigenesis in many cancer types including colon. However, the role of YAP1 in promoting colon tumorigenesis remains unclear. Here, we demonstrate that YAP1 expression is associated with M2 tumor-associated macrophage polarization and the generation of colon cancer stem-like cells. YAP1 downregulation by gene silencing or a phytochemical, ovatodiolide, not only suppresses colon cancer tumorigenesis but also prevents M2 TAM polarization.

**Methods:**

Human monocytic cells, THP-1, and colon cancer cell lines, HCT116 and DLD-1, were co-cultured to mimic the interactions between tumor and its microenvironment. M2 polarization of the THP-1 cells were examined using both flow cytometry and q-PCR technique. The inhibition of YAP1 signaling was achieved by gene-silencing technique or ovatodiolide. The molecular consequences of YAP1 inhibition was demonstrated via colony formation, migration, and colon-sphere formation assays. 5-FU and ovatodiolide were used in drug combination studies. Xenograft and syngeneic mouse models were used to investigate the role of YAP1 in colon tumorigenesis and TAM generation.

**Results:**

An increased YAP1 expression was found to be associated with a poor prognosis in patients with colon cancer using bioinformatics approach. We showed an increased YAP1 expression in the colon spheres, and colon cancer cells co-cultured with M2 TAMs. YAP1-silencing led to the concomitant decreased expression of major oncogenic pathways including Kras, mTOR, β-catenin, and M2-promoting IL-4 and tumor-promoting IL-6 cytokines. TAM co-cultured colon spheres showed a significantly higher tumor-initiating ability in vivo. Ovatodiolide treatment alone and in combination with 5-FU significantly suppressed in vivo tumorigenesis and less TAM infiltration in CT26 syngeneic mouse model.

**Conclusions:**

We have identified the dual function of YAP1 where its suppression not only inhibited tumorigenesis but also prevented the generation of cancer stem-like cells and M2 TAM polarization. Ovatodiolide treatment suppressed YAP1 oncogenic pathways to inhibit colon tumorigenesis and M2 TAM generation both in vitro and in vivo. Ovatodiolide should be considered for its potential for adjuvant therapeutic development.

**Electronic supplementary material:**

The online version of this article (doi:10.1186/s13045-017-0421-3) contains supplementary material, which is available to authorized users.

## Background

Colorectal cancer (CRC) represents one of the most prevalent malignancies in the world with an estimated 9% of all cancer incidence [1]. The pathogenesis of CRC is complex due to many factors including environmental risk factors, inherited genetic risks, and nutritional practices [2]. The development of most CRC cases are sporadic from dysplastic adenomatous polyps. These are the results of several oncogenes such as KRas, c-myc, β-catenin, and other less frequent but also powerful tumorigenic pathways [3, 4]. Besides these genetic abnormalities, the formation of an inflammatory microenvironment also plays an essential role for CRC progression. Tumor-associated macrophages (TAMs) as one of the most abundant tumor-infiltrating stromal cell types have been shown to promote metastasis, drug resistance, and associated with a poor prognosis in CRC patients. Recent studies suggest an intimate association between the generation and/or maintenance of cancer stem cells (CSCs) and TAMs [5]. The presence of CSCs potentiates the epithelial-to-mesenchymal transition (EMT), treatment resistance, and tumor repopulation [5]. Thus, targeting TAMs represents an important milieu for anti-cancer drug development.

Yes-associated protein 1 (YAP1) is a well-established oncogenic factor in hepatocellular carcinoma and recently indicated in colorectal cancer [6–8]. YAP1 was proposed to functioning either partially or in tandem with key oncogenic drivers such as Kras, β-catenin, and Akt/mTOR in colon tumor initiation and progression [6, 8, 9]. However, the role of YAP1 and the tumor microenvironment remains unclear. In this study, we investigated the role of YAP1 in promoting colon tumorigenesis in tumor cells and association with M2 TAM polarization. We hypothesize that oncogenic YAP1 promotes the generation of M2 TAM polarization and in return M2 TAM further enhances tumorigenic phenotypes including enhanced proliferation, colony formation, colon-sphere generation, and metastatic potential. Therefore, agents that target and suppress the expression of YAP1-mediated signaling may act as a dual anti-cancer agent by disrupting the molecular connections between TAMs and tumor cells.

Ovatodiolide (OV) is a bioactive phytochemical purified from *Anisomeles indica* (L.) Kuntze (Labiatae), a medicinal herb which has been widely used for the treatment of a variety of inflammation-associated diseases and implicated for potential anti-cancer functions by our previous reports [10, 11]. Various oncogenic targets inhibited by ovatodiolide have been implicated; these include TNF-α, NF-κB, β-catenin, MMPs, and others to achieve anti-cancer effects such as the induction of cell cycle arrest, apoptosis, and reduction of metastatic potential [12–15]. Since the connection between inflammation (TAMs) and colon tumorigenesis has been well established, we aimed to examine the potential functions of ovatodiolide (being an anti-inflammatory and anti-cancer agent) in targeting both tumor cells and TAMs.

## Results

### Increased YAP1 expression is associated with poor prognosis

**Fig. 1:**
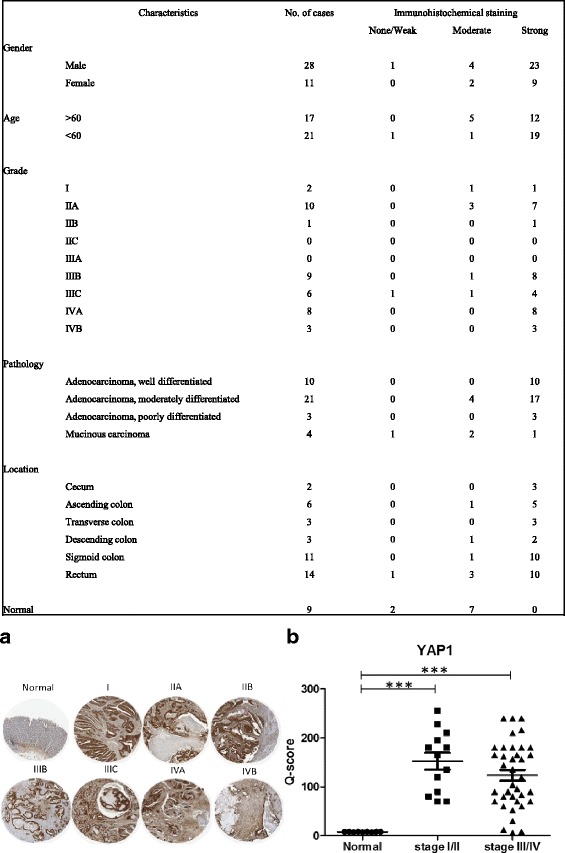
Increased YAP1 expression is associated with colon cancer malignancy. **a** Using public database, we first found that YAP1 mRNA expression is elevated (at least 2-fold increase) in colorectal adenoma samples as compared to normal tissues (Sabates-Bellver database). **b** Another database (Kaiser Colon database) indicated that YAP1 mRNA was significantly increased in a cohort of colon adenocarcinoma samples. **c** An increased YAP1 protein expression profile was supported by clinical sample database (*i*, Antibody CAB009370, ProteinAtlas.org); *ii*. Our immunohistochemical analysis also showed that YAP1 expression in normal colon crypts and a significantly increased YAP1 expression was found in the rectal adenocarcinoma section. The *number* indicates the area of magnified view. **d** YAP1 expression was also found to be associated significantly to time of disease recurrence [19]. **e** Patients with higher risk for colon cancer demonstrate both high YAP1 and IL-6 expression (*upper panel*); patients with combined high expression of IL-6 and YAP1 were found to be associated with a significantly lower specific survival rate (Colon Metabase, SurvExpress). *P* values are indicated in each panel

**Fig. 2:**
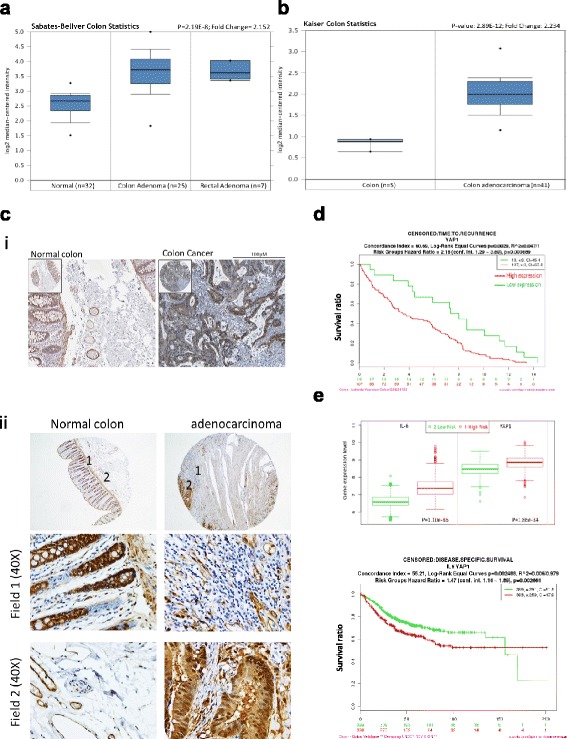
YAP1 expression profile in colon cancer tissue microarray. A significantly increased staining of YAP1 was predominately found in the colon cancer samples. Strong staining (*Q* score ≥150) was found in stages IIIB, IIIC, IVA, and IVB suggesting that YAP1 expression may be associated with the progression of colon CA. Total number of samples (cancer 39 and normal tissues 9). **a** Representative micrographs of the tissue microarray are shown (stained with YAP1 antibody). **b** Quantitative analysis of YAP1 staining. The *Q* score of each sample was plotted and showed that strong YAP1 staining was associated in the tumor samples as compared to the normal tissues. ***P < 0.001. Scoring analysis was performed using *Q* score as established previously [49]

We explored the public databases to show that YAP1 network is upregulated in clinical colon cancer samples and cell lines. First, in Sabates-Bellver colon database [16], composing of 64 clinical samples from different regions, YAP1 messenger RNA (mRNA) was significantly elevated in both colon and rectal adenoma samples (Fig. 1a). In Kaiser colon database analysis (Fig. 1b), comparing normal colon tissues and colon adenocarcinoma, we found that YAP1 mRNA was approximately 2.2-fold higher in the adenocarcinoma samples [17]. Consistently, an elevated YAP1 immunostaining was detected in the paraffin-fixed sections from a small cohort of colorectal cancer patients (Right panel, Fig. 1c, i). In normal colon tissue sections, YAP1 staining appears to be low to medium in the epithelial and glandular cells and low in the nerve/peripheral ganglion (Left panel, Fig. 1c, i) (images obtained from ProteinAtlas database, CAB009370). Our tissue microarray analysis supported that YAP1 expression was found in normal colon crypts and a significantly increased signal was identified in the rectal adenocarcinoma section (Fig. 1c, ii). Consistently, using the online bioinformatics tool SurvExpress, with the default setting, a higher YAP1 expression was associated with a significantly shorter time of recurrence (Fig. 1d) [18]. In addition, the IL-6/YAP signaling has been shown to be associated with increased stemness in breast cancer [19]. In the same database, patients with high risk of recurrence showed a significantly higher IL-6 and YAP1 mRNA level (upper panel, Fig. 1e); in a larger database (Colon Metabase, SurvExpress), a higher expression of IL-6 and YAP1 predicts a lower survival rate (lower panel, Fig. 1e). In support, using a colon tissue microarray, we identified a strong YAP1 expression in the late stages (stages IIIB, IIIC, IVA, and IVB) colon cancer samples (Fig. 2). Furthermore, a recent clinical study demonstrated that YAP1 activation was associated with the poor prognosis and certuximab resistance in colon cancer patients [20]. Collectively, these findings support our hypothesis that YAP1 represents a key target for therapeutic development in colon cancer patients.

### Tumor-associated macrophages (TAMs) promote the generation of CD133+ and side-population stem-like cells and associated with increased YAP expression

**Fig. 3:**
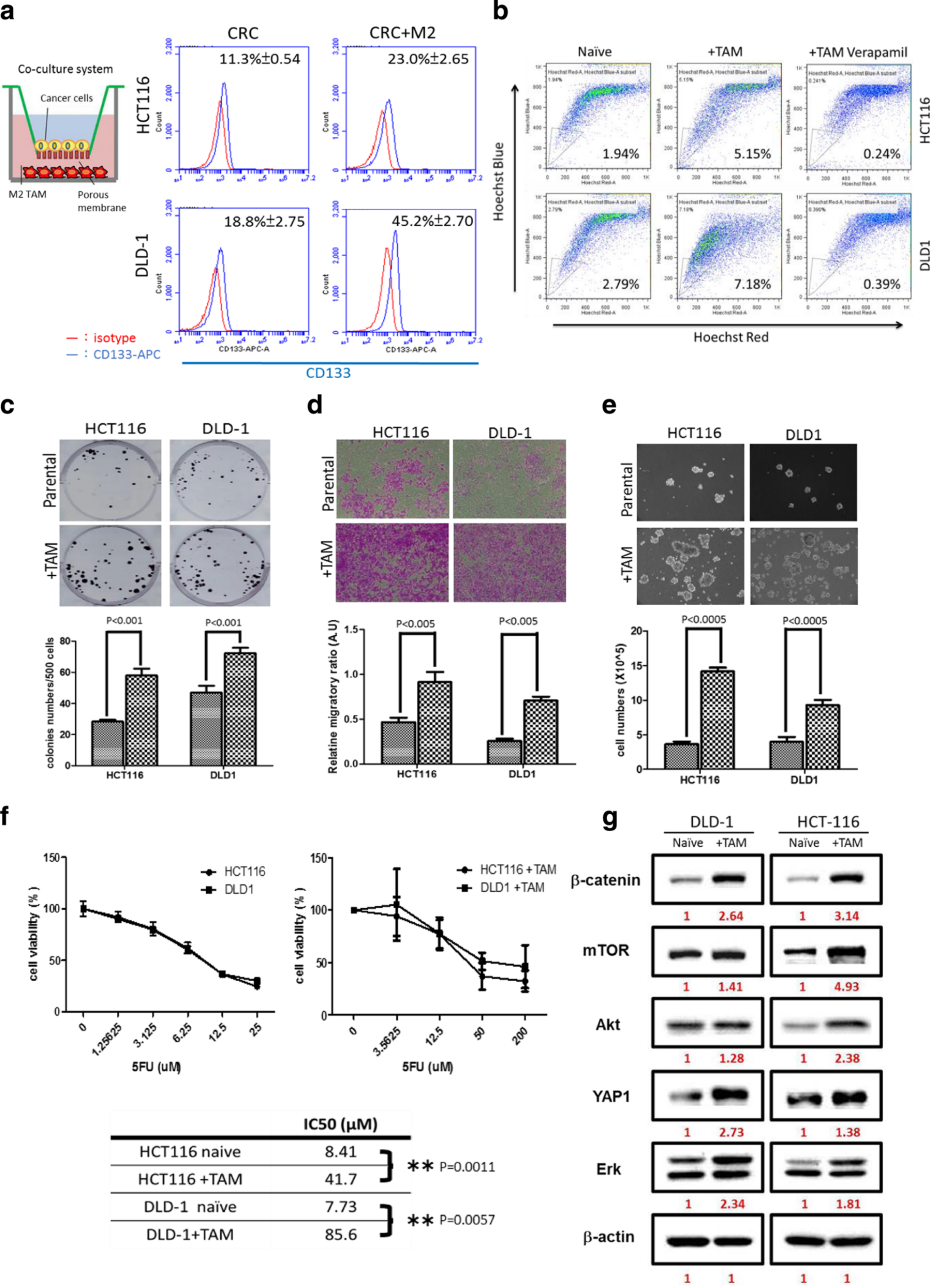
M2 TAM promotes colon tumorigenesis associated with increased YAP1 and associated oncogenic pathways. M2 TAM co-culture increased CD133+ (**a**) and side-population (**b**) in HCT116 and DLD-1 cells, respectively. A representative side-population analysis is shown here. M2 TAM also enhanced the colony-forming (**c**) and migratory (**d**) abilities in both HCT116 and DLD-1 cells. **e** Increased sphere formation. **f** SRB analysis demonstrates a significant increase in the 5-FU resistance of the TMA co-cultured HCT116 and DLD-1 cells (*right panel*) as compared to their naïve counterparts (*left panel*). The comparative IC_50_ values are listed in the table below (**g**) The enhanced tumorigenic ability is associated with an elevated expression of oncogenic markers YAP1, Akt/mTOR, Erk, and colon stemness maker, β-catenin. The *number* beneath each band represents the expression ratio of each signal normalized against actin (as 1). All experiments were done in triplicates

Tumor-associated macrophages (TAMs) are implicated in the development and progression of colorectal cancer [21]. Here, we investigate the role of TAMs in generating drug-resistant CRC cells. Both DLD-1 and HCT116 cells were co-cultured with TAMs and subjected to flow cytometric analysis. We found that in the presence of TAMs, the percentage of CD133+ (Fig. 3a) and side-population cells (Fig. 3b) in both DLD-1 and HCT116 were significantly increased. TAM co-cultured CRC cells showed markedly increased colony-forming (Fig. 3c), migratory (Fig. 3d), and self-renewal (Fig. 3e) abilities as compared to their naïve counterparts. The increased tumorigenic phenotypes by TAM co-culture was associated with a higher expression in YAP1, ERK, Akt/mTOR (oncogenic makers), and β-catenin (both oncogenic and stemness marker) (Fig. 3g). More importantly, TAM co-culture resulted in increased resistance toward 5-FU treatment (Fig. 3f). For instance, in the presence of TAM, the IC50 value of 5-FU of DLD-1 was approximately 11-fold higher than its naïve counterpart (85.6 versus 7.73).

### TAM educated CRC cells showed enhanced tumor-initiating ability

**Fig. 4:**
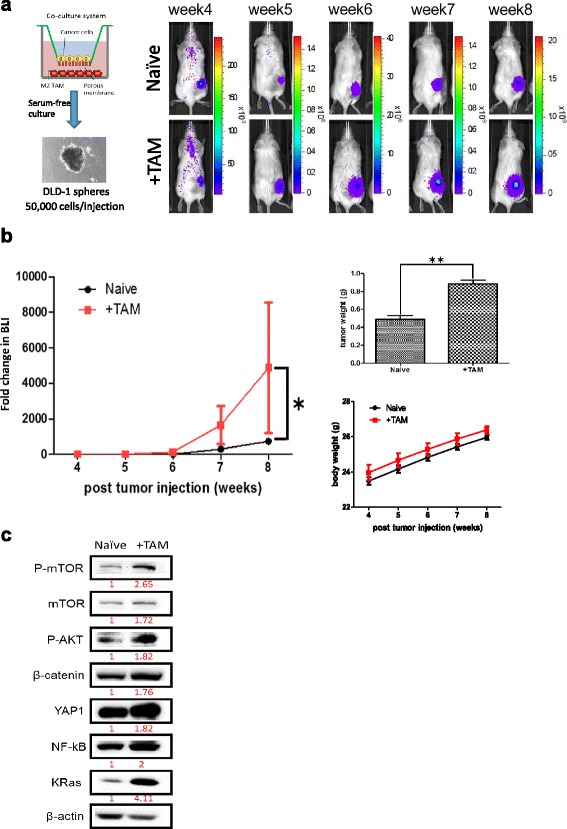
TAM co-culture increased tumor-initiating ability in vivo. **a** DLD-1 cells co-cultured with M2 TAMs were subsequently cultured under serum-deprived conditions to generate DLD-1 spheres. DLD-1 + TAM group initiated tumor growth significantly faster than its naïve counterparts. Representative bioluminescence images are shown (*N* = 5 per group). **b** Semi-quantitative bioluminescence comparison between DLD-1 + TAM and naïve groups. Fold change in tumor size was indicated by the fold change in bioluminescence intensity over time. Tumor growth (represented as fold change in bioluminescence intensity, BLI) was significantly higher in the DLD-1 + TAM group, *P < 0.05. The *upper right panel* shows the significantly higher average tumor weight collected from DLD-1 + TAM group as compared to its naïve counterparts, **P < 0.01. The *lower right panel* shows body weights of both groups over time. **c** Western blot analysis of tumor biopsies indicated that DLD-1 + TAM sample exhibited a markedly increased YAP1, Akt/mTOR, NF-kB, and Kras signaling. The expression intensity was compared using arbitrary ratios normalized against β-actin (as 1)

TAM-enhanced tumor-initiating ability was tested in vivo. Tumor spheres generated from naïve DLD-1 and from TAM co-cultured DLD-1 (termed + TAM) were injected subcutaneously into NOD/SCID mice to evaluate their tumor-initating potential. We found that the+TAM group initiated tumor growth significantly faster as compared to their naïve counterparts (Fig. 4a). Fold change in tumor size was measured semi-quantitatively based on the bioluminenesce signals; a significantly heavier tumor weight was found in the+TAM group, reflecting the increased tumorigenesis (Fig. 4b). In agreement with our in vitro data, +TAM tumor sample showed an increased level of YAP1, Kras, Akt/mTOR, NF-kB (oncogenic markers), and β-catenin (both oncogenic and stemness marker, Fig. 4c).

### Ovatodiolide treatment suppresses tumorigenesis in CRC cells

**Fig. 5:**
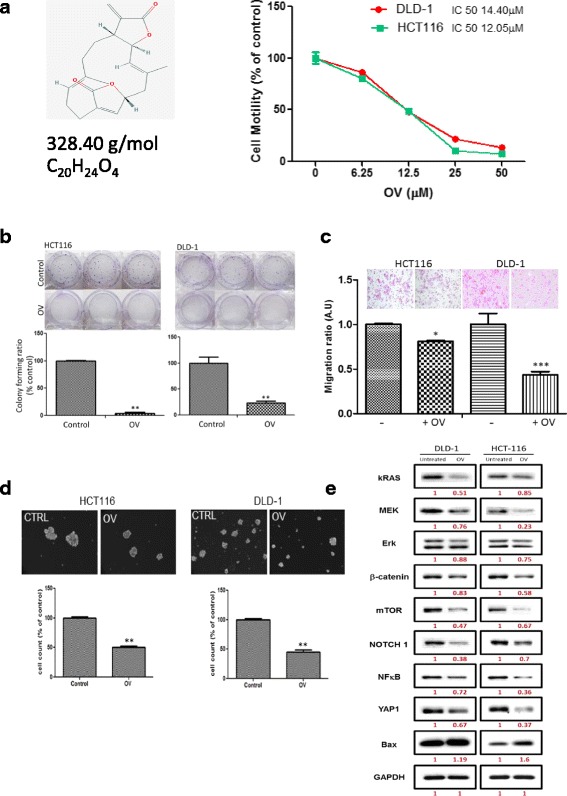
Ovatodiolide treatment suppresses CRC tumorigenesis and colon sphere generation. **a** Cell viability assay (SRB) demonstrates that ovatodiolide is effective in suppressing cell viability in CRC cell lines. The IC50 values are indicated. The *insert* depicts the 2D chemical structure of OV (diagram adapted from PubChem CID 6451060; molecular formula C20H24O4; molecular weight 328.40216 g/mol). OV (OV 2.8 μM approximately IC_10_) effectively suppresses colony-forming (**b**), migratory (**c**), and self-renewal (**d**) abilities of both HCT116 and DLD-1 CRC cell lines. **P < 0.01. **e** OV-mediated inhibitory effects on CRC cells were associated with the decreased expression of YAP1, Kras/MEK/ERK, NF-kB, mTOR (oncogenic markers), β-catenin, Notch1 (stemness markers), and increased Bax (apoptosis marker). OV treatment (14 μM, 48 h, 1 × 10^6^ HCT116 and DLD-1 cells). The *number* beneath each band represents the expression ratio of each signal normalized against actin (as 1)

We intend to identify compounds which not only target cancerous cells but also TAMs, in contrast to the conventional chemotherapeutic agents which only aim to eliminate cancerous cells. Previously, studies from us and others showed ovatodiolide (OV) as an anti-inflammatory and anti-cancer agent in different cell models [10, 22, 23]. Here, we first demonstrated that OV effectively inhibited the cell viability of HCT116 and DLD-1 colon cancer cells (Fig. 5a). In addition, OV treatment significantly inhibited the colony formation (Fig. 5b) and migration (Fig. 5c). We subsequently showed that OV treatment suppressed the self-renewal ability of CRC cells under serum-deprived condition as reflected by the significantly lower number of colon spheres formed (Fig. 5d). OV-mediated anti-CRC effects were found in association with the decreased expression of YAP1, Kras/MEK/ERK, NF-kB, mTOR (oncogenic markers), β-catenin, and Notch1 (stemness markers) while an increased in Bax expression (pro-apoptosis marker).

### Ovatodiolide treatment suppresses M2 polarization in vitro

**Fig. 6:**
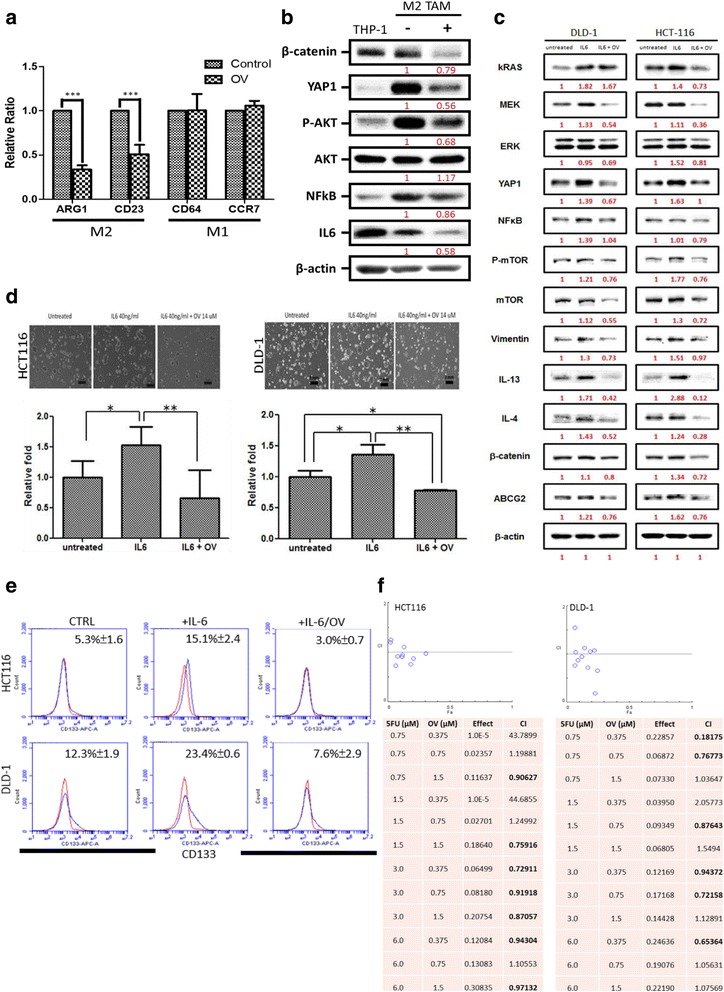
Ovatodiolide treatment suppresses the M2 TAM polarization. **a** Macrophage progenitor cells, THP-1 cells, were cultured under M2 macrophage conditional medium with and without the treatment of ovatodiolide (OV, 14 μM, 48 h). Real-time PCR analysis demonstrated that the addition of OV significantly decreased the M2 markers including ARG1 and CD23 while not affecting M1 polarization in the differentiated macrophages. **b** Western blot analysis of OV-treated macrophages demonstrated decreased YAP1, NF-kB, phosphorylated-Akt/Akt, and IL-6. THP-1 served as a control for M2 TAMs. The *minus sign* indicates M2 TAMs without OV treatment while the *plus sign* indicates M2 TAMs treated with OV (14 μM, 48 h). **c** IL-6 addition simulates the presence of M2 TAMs promoted colon tumorigenic profiles including enhanced Kras, b-catenin, YAP1, NF-kB, and Atk/mTOR signaling; the addition of OV significantly reversed IL-6 induced tumorigenic profiling. **d** The addition of IL-6 markedly enhanced the formation of colon spheres in both cell lines, and the OV treatment suppressed it. **e** OV treatment markedly reduced IL-6-induced CD133+ cell population in both HCT116 and DLD-1 cell lines. **f** The combination index (CI) analysis showed that 5-FU and OV synergistically inhibited both DLD-1 and HCT116 cell viability. Synergism (CI < 1, shown in *bold* font), additive effect (CI = 1), and antagonism (CI > 1). All experiments were performed in triplicates

After establishing ovatodiolide’s anti-CRC effects, we examined if it could also prevent the generation of M2-polarized tumor-associated macrophages (M2-TAMs), known to promote tumor progression in many cancer types including colon [24]. Human monocytic THP-1 cells were used as the macrophage precursor for our study. Ovatodiolide treatment prevented M2 polarization in THP-1 cells (Fig. 6a), as reflected by the significantly decreased M2 markers (ARG1 and CD23) while no significant difference in M1 markers (CD64 and CCR7). We then demonstrated that ovatodiolide treatment led to the decreased expression of YAP1, β-catenin, Atk, and NF-kB in the M2 TAMs as compared to their un-treated counterparts (Fig. 6b). One of the major cytokines secreted by M2-TAM is IL-6, and IL-6’s oncogenic roles have been well established in different cancer types including colon cancer [25, 26]. Notably, M2 TAMs express an elevated level of IL-6, and ovatodiolide treatment significantly decreased IL-6 expression (Fig. 6b).

To explore if ovatodiolide treatment can suppress IL-6-induced colon carcinogenesis, exogenous IL-6 was introduced to the culture medium. We showed that the addition of IL-6 significantly increased the expression of multiple oncogenic markers including Kras-MEK, YAP1, NF-kB, vimentin (EMT marker), β-catenin (a marker for both oncogenesis and stemness), and ABCG2 (drug resistance) in both HCT116 and DLD-1 cells. Ovatodiolide treatment significantly suppressed these IL-6-induced pathways (Fig. 6c). For instance, major pro-M2 TAM polarization cytokines, IL-4 and IL-13 expression, were markedly suppressed by the OV treatment even in the presence of IL-6 (Fig. 6c).

IL-6 treatment significantly induced the generation of colon spheres (Fig. 6d), and the addition of OV markedly reduced this effect (Fig. 6d). IL-6-enhanced tumor sphere-forming ability was disrupted significantly as the size and the number of formed spheres was significantly less comparing to IL-6-induced counterparts (Fig. 6d). In support, exogenous IL-6 resulted in an increase in CD133+ HCT116 and DLD-1 cells. The addition of ovatodiolide (in the presence of IL-6) significantly reduced the IL-6-induced effects (Fig. 6e). Furthermore, we tested the possibility of ovatodiolide working in synergy with 5-FU to suppress the viability of colon cancer cells. Different concentration combinations of ovatodiolide and 5-FU were assayed to generate the isobolograms. We found that several combinations of 5-FU and ovatodiolide inhibited both HCT116 and DLD-1 cell viability synergistically (CI index <1, in bold, Fig. 6f).

### YAP1-silencing led to decreased CRC tumorigenesis and M2 polarization

**Fig. 7:**
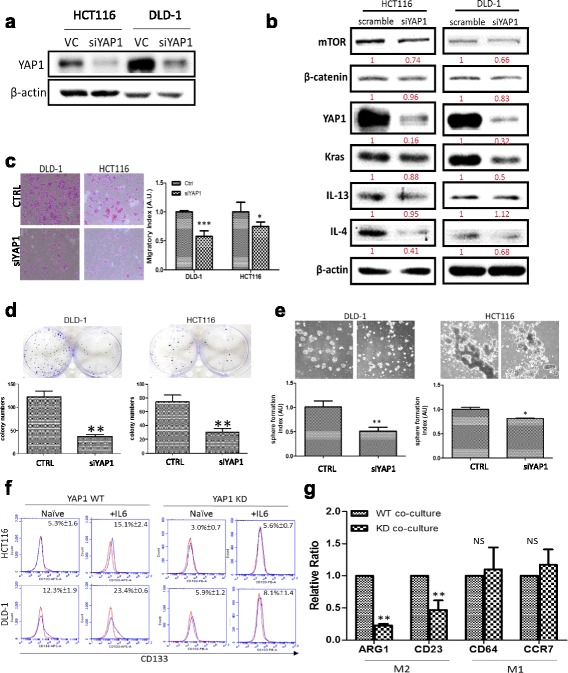
YAP1 silencing significantly decreased CRC tumorigenesis and M2 TAM generation. **a** Western blot analysis demonstrated YAP1 downregulation using siRNA versus vector control (VC). **b** YAP1 silencing in both DLD-1 and HCT116 cells were associated with a marked decreased expression of several key oncogenic pathways, Kras, NF-kB, mTOR, and β-catenin as well as the pro-M2 cytokine IL-4. **c**–**e** YAP1-silencing also led to the decreased migratory, colony-forming, and sphere-generating abilities in both cell lines, respectively. **P < 0.01, *P < 0.05. **f** Flow cytometric analysis showed YAP1-silencing significantly reduced the percentage of CD133+ cells in both cell lines even with the exogenous IL-6 stimulation. **g** Comparative M2 polarizing ability between THP-1 macrophages co-cultured with YAP1 wild-type (WT) and YAP1-knockeddown (KD) DLD-1 cells. Real-time PCR analysis demonstrates that the M2-polarization process was significantly reduced in THP-1 cells (co-cultured with YAP1-KD DLD-1 cells) as indicated by the significantly reduced M2 markers (ARG1 and CD263) while M1 phenotype was not affected. NS denotes not significant

Subsequently, YAP1 expression was downregulated using siRNA technique in both HCT116 and DLD-1 cells (Fig. 7a); stemness markers (YAP1, β-catenin), oncogenic markers (Kras, Akt/mTOR), drug resistance marker (ABCG2), pro-M2 polarization cytokines (IL-4 and IL-13), and mesenchymal marker (vimentin) were downregulated in the wake of YAP1 silencing. These observations were comparable to those found in OV treatment (Fig. 6). The tumorigenic phenotypes including colony formation (Fig. 7b), migration (Fig. 7c), sphere formation (Fig. 7d), and resistance against 5-FU (Fig. 7e) were significantly suppressed in YAP1-silenced cells. In addition, the percentage of CD133+ cells were also markedly reduced in the YAP1-silenced cells; more importantly, YAP1-silencing led to a less IL-6-responsive cells as indicated by the lower percentage of CD133+ cells under the exogenous IL-6 treatment (Fig. 7f). When co-cultured with YAP1-silenced DLD-1 cancer cells, THP-1 monocytes differentiated into macrophages expressing a significantly reduced M2 marker. For instance, M2 markers such as ARG1 and CD23 were significantly lower than the THP-1 macrophages differentiated under YAP1 wild-type DLD-1 cells (Fig. 7g). Interestingly, the M1 markers such as CD64 and CCR7 did not appear to be significantly affected. In support, THP-1 macrophages co-cultured with YAP1-overexpressing DLD-1 or HCT116 cells resulted in an increased expression of M2 markers (Additional file 1: Figure S2B).

Equally important, the number of CD133+ cells were significantly increased in both YAP1-overexpressing DLD-1 and HCT116 cells (Additional file 2: Figure S1A); the tumor sphere-forming ability was also markedly enhanced in YAP1-overexpressing cells (Additional file 2: Figure S1B). In consistent with our proposed role for YAP1, when YAP1 expression is increased so is the level of β-catenin, NF-kB, vimentin, and Kras (Additional file 2: Figure S1C).

The role of YAP1 in M2 polarization was also tested in THP-1 cells by silencing YAP1 (Additional file 1: Figure S2A). We found that the resultant macrophages differentiated from YAP1-silenced THP-1 showed a significantly reduced level of IL-4, TGFB1, and Ym2 (M2 markers) while increased iNOs (M1 marker).

### OV treatment suppressed CRC tumorigenesis and M2 infiltration in vivo

**Fig. 8:**
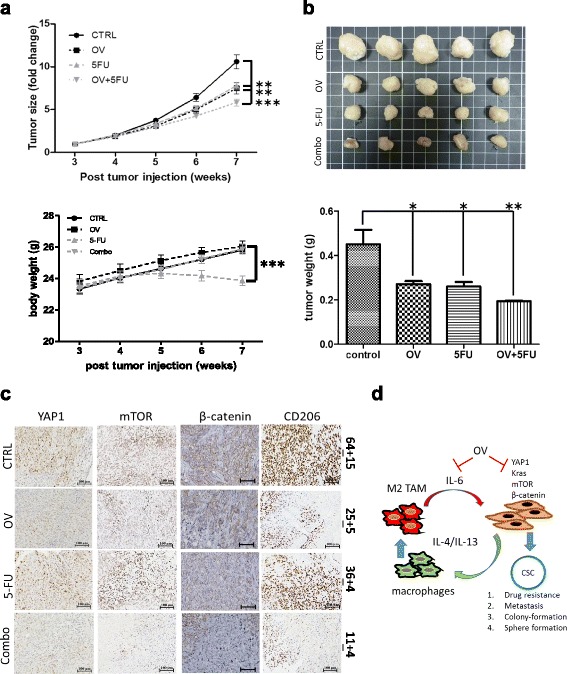
Ovatodiolide suppressed tumorigenesis and macrophage infiltration in vivo. **a** In vivo evaluation of tumor growth suppressive effect. Ovatodiolide (5 mg/kg, five times/week), 5-FU (30 mg/kg, two times/week), and combination regimen were evaluated. Tumor inhibitory effect was the most significant in the combination group followed by OV and 5-FU alone groups. The bodyweight of the animals were not negatively affected except for the 5-FU group. **b** Photographs and average weight of tumor samples collected from each group. **c** Immunohistochemical staining of tumor sample sections. Oncogenic YAP1, mTOR, and β-catenin and CD206 (M2 marker) expression was most significantly suppressed in the combination group followed by OV and 5-FU group. The *numbers* indicate the average number of CD206+ cells (three random fields were counted and averaged). **d** Schematic diagram illustrating the anti-CRC effect by OV treatment is 2-fold, targeting both oncogenic pathways in colon cancer cells and the generation of M2 TAMs in the microenvironment

The potential colon cancer suppressive effect of ovatodiolide was investigated in vivo using immune competent mouse xenograft model with CT26 mouse colon cancer cells. OV treatment (5 mg/kg, five times/week) appeared to be as effectively in suppressing tumor growth as standard chemotherapeutic agent 5-FU (30 mg/kg, twice a week) while the combined treatment yielded the most significant tumor inhibitory effect (Fig. 8a, b). Immunohistochemical analysis of the tumor samples revealed that OV alone and OV + 5FU exhibited less number of M2 TAM, as reflected by the lower number of CD206+ cells as compared to those in control tumor counterparts (Fig. 8c); the tumor growth inhibitory effect of OV and OV + 5-FU may be attributed to the reduced staining of YAP1, mTOR, and β-catenin.

## Discussion

Tumor-associated macrophages or TAMs (the M2-polarized TMAs particularly) have been indicated to promote tumorigenesis and drug resistance [27, 28]. The increased abundance of tumoring infiltrating M2 TAMs has been correlated with poor prognosis in various types of human cancers including colon [24, 29, 30]. The modification and/or disruption between the molecular communications between colon cancer cells and its microenvironment (TAMs) thus represents an important milieu for intervention development. Here, we first demonstrated that the presence of M2 TAMs promotes the generation of cancer stem-like phenotypes in both HCT116 and DLD-1 colon cancer cells; M2 TAM co-cultured cancer cells demonstrates increased CD133 expression, colon sphere-forming ability in association with a significantly resistant 5-FU resistance.

M2 TAMs have been shown to promote tumorigenesis via the secretion of a plethora of cytokines [31]. One of the major M2 TAM-secreted oncogenic cytokines, IL-6, has been implicated in contributing metastatic potential and therapeutic interventions in different cancer types including colon [26, 32]. Our study adds another dimension of IL-6’s role in colon tumorigenesis where IL-6 from M2 TAMs or exogenous source enhanced the generation of colon stem-like cells (or colon spheres); the addition of IL-6 was associated with an increased expression of several oncogenic pathways including YAP1, Kras, β-catenin, NF-κB, and mTOR and the increased expression of IL-4 and IL-13, both of which have been established for the promotion of M2 phenotype [33].

Experimental and clinical evidence indicate that targeting and eliminating TAMs represent a potential therapeutic strategy of cancer therapy [34, 35]. However, previous studies suggested that targeting M2 TAMs systemically carries the risk of compromising the host’s immunity [27, 36, 37]. Here, we identified YAP1 as a potential molecule whose expression is associated with M2 polarization and colon tumorigenesis. First, an increased YAP1 expression was detected in colon spheres and M2 TAMs. Second, the downregulated YAP1 expression in colon cancer cells by either gene-silencing technique or phytochemical treatment of ovatodiolide led to the decreased tumorigenesis (colony formation, migration, and tumor sphere formation) accompanied by the decreased M2-polarizing cytokines such as IL-4 (the major one affected by YAP1 silencing) and IL-13 (to a lesser extent) as well as mTOR/Akt signaling. Our observation was supported by a study indicating that IL-4/IL-13/Akt [38–40] signaling axes are important for M2 phenotype generation.

In parallel, ovatodiolide treatment suppressed YAP1 along with other major oncogenic pathways and prevented the THP-1 monocytes from M2 polarization in association with a decreased oncogenic cytokine IL-6 expression. Notably, IL-6 has been shown to contribute to proliferation and promote EMT and self-renewal in breast cancer cells [27] establishing an essential communication between cancer cells and M2 TAMs. We showed that exogenous IL-6 led to the increased generation of colon tumor spheres with concomitant elevated YAP1 expression, establishing a novel link between YAP1 and IL-6 network. Moreover, bioinformatics analysis (data not shown) indicated that downregulation of YAP1 is associated with a decreased level of IL-10RB (Interleukin-10 receptor subunit beta) implicating their collaborative roles in M2 polarization process. Together, our data suggests that YAP1’s function in promoting colon tumorigenesis not only derives from its intrinsic oncogenic properties but also functions to promote M2 TAM polarization. Interestingly, previous report indicated that oncogenic Kras, YAP1, and β-catenin serve similar functions in cell cycle control in tumor initiation [8]. A recent seminal study showed that acquired resistance against Kras inhibition in a Kras-driven mouse lung cancer model was associated with an increased YAP1 signaling; Kras and YAP1 signaling converges and activates the cellular EMT machinery [41]. This is in agreement of our finding that YAP1 suppression either by ovatodiolide or gene silencing significantly reduced the expression of Kras and oncogenic properties (including sphere-forming ability).

The anti-cancer and self-renewal properties of ovatodiolide have been previously established by our group [10, 15, 22]. The present study furthered the exploration of ovatodiolide’s anti-cancer function by showing its effects on preventing the generation of M2 TAMs. In this study, we identified the elevated YAP1 expression in M2 TAMs and ovatodiolide treatment prominently suppressed the expression of YAP1, M2 markers (ARG1 and CD23), and inflammatory-signaling networks such as NF-κB and Akt. Our findings suggest that YAP1 within the tumor cells may function in promoting colon tumorigenesis via its association with aforementioned oncogenic pathways and generating cancer stem-like cells (as shown in our study) while generating M2 TAMs via increased M2-polarizing IL-4 and IL-13 cytokines into the stroma (Fig. 8d for proposed model). In addition, ovatodiolide-mediated IL-6 suppression in the M2 TAMs represented another important anti-cancer attribute of ovatodiolide; even in the presence of exogenous IL-6 which increased the generation of cancer stem-like cell population in both HCT116 and DLD-1 cell lines, ovatodiolide was able to attenuate IL-6’s effects.

Certainly, ovatodiolide-mediated anti-cancer effects in this study appeared to be multi-targeted and differential between HCT116 and DLD-1 cells. This reflects the heterogeneity of cancer and the importance of precision medicine. HCT116 and DLD-1 cells used in this study share a very similar gene mutation profile. But one of the major difference is that DLD-1 is a TP53 mutant while HCT116 wild-type. This may explain that HCT116 is slightly more sensitive toward ovatodiolide’s treatment; HCT116 also contains a lower intrinsic YAP1 expression level as compared to DLD-1 and demonstrates a lower ability to form tumor spheres as compared to DLD-1. A recently study indicated that primary breast cancers harboring TP53 mutations and expressing high level of YAP1 demonstrate a higher expression level of cyclin A, cyclin B, and CDK1 genes as compared to the TP53 wild-type counterparts [42]. It warrants further investigations to determine if this phenomenon also exists in colon cancer and for establishing a colon cancer signature for ovatodiolide sensitivity.

## Conclusions

Here, we demonstrated for the first time that YAP1 played a key role in colon tumorigenesis in a dual dimensional fashion where it acts in cancer cells to promote the malignant phenotypes including cancer stem-cell like properties and in polarizing TAMs toward M2 phenotype. The suppression of YAP1 either by siRNA or ovatodiolide suppressed YAP1-associated oncogenic characteristics. Thus, YAP1 could represent an important druggable target; ovatodiolide could be further evaluated and considered to be used as an adjuvant agent for treating colon cancer.

## Methods

### Cell culture and chemicals

The human colon cancer cell lines HT-29, SW480, DLD-1, HCT116, and monocytic cell line, THP-1, were obtained from the American Type Culture Collection (ATCC), and cells were cultured according to ATCC’s recommended conditions. Colon sphere formation assay was performed according to previously established method [43]. DLD-1 and HCT116 cells were cultured in Serum-Free Medium (SFM) composed of DMEM/Ham’s F12 (1:1), human epidermal growth factor (hEGF, 20 ng/ml), basic fibroblast growth factor (bFGF; 10 ng/ml (PeproTech, NJ, USA), 2 μg/ml of 0.2% heparin (Sigma), and 1% penicillin/streptomycin (P/S, 100 U/ml, Hyclone). Cells were seeded (1000 cells/ml) in 12-well low adhesion plates and incubated at 37 °C and 5% CO2 for 5–7 days. Cell aggregates or spheroids (compact, spherical, non-adherent masses >50 μM in diameter) were counted. 5-FU was purchased from SelleckChem, Taiwan (Cat. No.S1209), and ovatodiolide was isolated and purified as described previously [44].

### Cell viability determination

Cellular viability in this study was determined using the sulforhodamine B (SRB) assay [45]. Briefly, colon cancer cells and/or spheres were seeded in 96-well plates (3.5 × 10^5^ cells/well) and treated with drugs of interest (ovatodiolide or 5-FU) alone or in combination at indicated concentrations and times. Post treatment, the relative cell number was determined using a SRB reagent according to the manufacturer’s protocol (Sigma, USA). For tumor spheres or non-attached cells, the cell viability was quantified using Alamar blue staining (Life Technologies, USA).

### Macrophage generation and differentiation

Macrophage generation and differentiation from THP-1 cells were performed according to previously established method [46]. In brief, for M2-polarized macrophages, THP-1 cells were first treated with 320 nM PMA for 6 h, followed by cultured by the addition of IL-4 and IL-13 (20 ng/ml) for another 18 h. For M1-polarized THP-1 macrophages, LPS (100 ng/ml) and IFN-γ (20 ng/ml) were used instead. THP-1 cells were also co-cultured with CRC cell lines. CRC cells were seeded in the upper insert of a six-well Transwell apparatus (0.4 μM pore size, Corning, Lowell, MA) while THP-1 with 320 nM PMA in the lower chamber. After 48 h, both macrophages and CRC cells were then harvested for further biochemical analyses. In the colon cancer co-culture experiments, only PMA was added to the THP-1 cells (M0 macrophage progenitors) without the addition of IL-4 and IL-13. After 48 h of co-culture, both colon cancer cells and differentiated macrophages were harvested for further analyses.

#### Flow cytometry analysis

Flow cytometry was used to profile CRC stem-like cells using the BD Accuri™ C6 personal flow cytometer. CD133/1 (AC133) antibodies conjugated to APC (Miltenyi Biotec, Auburn, CA, USA) were used to determine CD133+ CRC cells. Side-population analysis was performed according to previously established method [47]. In brief, side-population HCT116 and DLD-1 cells were identified using FACSAria™ III sorter (BD Biosciences, Taiwan). Verapamil (100 μM final concentration) was added 15 min before Hoechst incubation and was used as a control. SP cells which express ATP-binding cassette ABCG2 and Hoechst 33342 efflux activity was identified and determined to be SP cells.

### Real-time PCR reaction

The total RNA was isolated and purified using TRIzol-based protocol (Life Technologies) according to the vendor’s instructions. Ten nanograms of total RNA were reverse transcribed using QIAGEN OneStep RT-PCR Kit (QIAGEN, Taiwan), and the PCR reaction was carried out using a Rotor-Gene SYBR Green PCR Kit (400, QIAGEN, Taiwan). The primer sequences for q-PCR experiments are as listed below:

**Table:** 

Gene	Forward	Reverse
AGR3	CATCACCTGGAGGATTGTCAATAC	TGAACTTATTCTGAGCCATTTCTTGT
CD23	GGGAGAATCCAAGCAGGAC	GGAAGCTCCTCGATCTCTGA
CD64	TGGGAAAGCATCGCTACAC	GCACTGGAGCTGGAAATAGC
CCR7	TCATTGCCGTGGTGGTAGTCTTCA	ATGTTGAGCTGCTTGCTGGTTTCG

(additional primer sequences may be found in Additional file 1: Figure S2).

### YAP1 silencing and overexpression

YAP1 expression was downregulated using siGNEOME SMARTpool YAP1 siRNAs (Catalog No. M-012200-00, Dharmacon). The gene-silencing experiments were carried out according to vendor’s instructions. After each gene-silencing experiment, western blots were used to verify that YAP1 gene product was successfully knocked down. YAP1 overexpression was performed by transfecting the cells with YAP1 open-reading frame vector (human, Cat. No. LV805163, Applied Biological Materials Inc., BC, Canada). The transfection protocol was performed by following vendor’s instructions.

### Western blot analysis

Total colon cancer cell lysates obtained from different experiments were separated using the SDS-PAGE using Mini-Protean III system (Bio-Rad, Taiwan) and transferred onto PVDF membranes using Trans-Blot Turbo Transfer System (Bio-Rad, Taiwan). The majority of the primary antibodies used in this study were listed. YAP1 (#4912), mTOR (#2983), and β-catenin (#9562) from Cell Signaling Techology (Taipei, Taiwan) and Kras (12063-1-AP), IL-6 (21865-1-AP), and IL-4 (16545-1-AP) from Protein Tech (Taipei, Taiwan). Secondary antibodies were from Santa Cruz Biotechnology (Santa Cruz, CA). Protein of interests were detected and visualized using ECL detection kit. Images were obtained and analyzed using UVP BioDoc-It system (Upland, CA, USA).

### Colony formation and migration assays

The colony-forming assay was performed using an established protocol [48] with modifications. Briefly, 500 HCT116 and DLD-1 cells were seeded in six-well plates with (2.8 μM ovatodiolide, equivalent of IC_10_ values) and without ovatodiolide. The plates were then stained using 0.005% crystal violet, and the colonies were counted. The cells were allowed to grow for another week. The cells were then harvested, fixed, and counted. The migratory ability of the cells was examined using Transwell migration assay (ThermoFisher, Taipei, Taiwan). In short, cells were trypsinized and re-suspended in a serum-free DMEM medium with or without treatments (siYAP or ovatodiolide of different dosing regimens). The cells were subsequently seeded into the upper chamber polycarbonate filters (8 μm pore size). A serum-containing DMEM medium (500 μL) was added to the lower chambers. The experiment was performed for either 24 or 48 h depending on the cell line. The cells were fixed with 3.7% formaldehyde and stained with crystal violet. The non-migratory cells on the upper side of the membrane were removed. The migratory ability was determined as the number of cells calculated on the lower side of the membrane.

### In vivo experiments

Female NOD/SCID and BALB/c mice were purchased from BioLASCO Taiwan Co., Ltd. The experiments were conducted strictly in compliance to the Affidavit of Approval of Animal Use Protocol Taipei Medical University (protocol LAC-2014-0170). First, the tumor-initiating ability test was performed using the tumor spheres generated from the naïve DLD-1 and M2 TAM co-cultured DLD-1 cells. Spheroids cells (1 × 10^5^ cells/injection) were subcutaneously injected into the right flank of NOD/SCID mice. DLD-1 expressing GFP and firefly luciferase dual reporter system (L2G, a generous gift from Dr. Sanjiv Sam Gambhir, Stanford University) was used for the bioluminescence imaging experiments. Tumorigenesis was monitored using bioluminescence (IVIS 200 system, Caliper) on a weekly basis and quantified using Living Imaging software. The tumor growth was indicated by the fold change in bioluminescence intensity plotted over time. Second, for drug treatment test and examination of TAM infiltration, subcutaneous tumor model was established using murine colon cancer cell line, CT26 (1 × 10^6^ cells/20 μL/injection) in BALB/c mice (4–6-weeks old). The treatments commenced when the tumor size reached approximately 100 mm^3^ measured by a standard caliper. Dosing regimens are as the following: 5-FU alone (30 mg/kg, two times/week), ovatodiolide alone (5 mg/kg, five times/week), and 5-FU + ovatodiolide combination (30 mg/kg, two times/week; 5 mg/kg five times/week, respectively). Both agents were given intraperitoneally. The change in tumor burden was expressed in fold change in cubic millimeter as compared to its starting volume. Mice were humanely sacrificed upon completion of experiment, and tumor biopsies were collected for further analyses.

### Immunohistochemical analysis

For preliminary screening, commercially available tissue microarray slides (Category # BA5 and CDA3) were purchased from SuperBioChips (Seoul, Korea). The staining protocol was followed as per the vendor’s published instructions (UltravVision Quanto Detection System HRP DAB manual, Thermo Sicentific, CA, USA). In short, the immunostaining was performed on 5-μm-thick tissue sections cut from the TMA (both commercially purchased and in vivo tumor samples). The sections were dewaxed and deparaffinized in xylene and rehydrated in graded alcohol solutions. Antigen-retrieval process was performed by heat-induction for 30 min in Tris-EDTA buffer. Slides were subsequently stained with primary antibodies of YAP1 (#4912, 1:400), mTOR (#2983, 1:500), β-catenin (#9562, 1:400, Cell Signaling technology, Taiwan), CD206 (anti-mannose receptor antibody, ab64693, 1: 400, Abcam, Taiwan), and their respective secondary antibodies. The sections were then counterstained with hematoxylin, followed by dehydration and mounting. The images were captured and recorded using Tissue FAXS viewer software (TissueGnotics, GmBH, Vienna, Austria).

### Statistical analysis

All experiments were performed and repeated at least three times. For in vitro experiments, bar charts and graphs represent mean values, and error bar indicates standard deviation (s.d). A paired “one-tailed” or ‘two-tailed” Student’s *t* test was performed using GraphPad Prism software. The result was considered significant when *P < 0.05, **P < 0.001, and ***P < 0.001, respectively.

## Additional files

Additional file 1: Figure S2. YAP1 expression is associated with M1-M2 polarization. (A) YAP1 silencing in macrophages (M0) leads to decreased M2 polarization. Real-time PCR analysis demonstrates that M2 markers, TGF-b1 and Ym2, were significantly reduced while one of the M1 marker iNOS was significantly increased, in the macrophages generated in the presence of DLD-1 cells. (B) YAP1-overexpressing colon cancer cells also promote the polarization of M2 genotype in THP-1 cells. Experiments were performed at least three times. **P* ≤ 0.05, ***P* ≤ 0.01, ****P* ≤ 0.001. The primer sequences used in the q-PCR experiments are shown. (PPTX 109 kb)
Additional file 2: Figure S1. YAP1 overexpression (OE) leads to increased stemness in colon cancer cells. (A) A significantly increased CD133+ cell percentage was found in the YAP1 OE cells as compared to their wild-type counterparts as demonstrated by our flow cytometric analysis. (B) YAP1-overexpressing HCT116 and DLD-1 cells were found to form a significantly higher number of tumor spheres (**P* ≤ 0.05, ***P* ≤ 0.01). (C) Comparative western blots of wild-type and YAP1 overexpressing HCT116 and DLD-1 cells. An increased expression of β-catenin, NF-kB, vimentin, and Kras is associated with YAP1 overexpression. (PPTX 396 kb)

## References

[1] Boyle P, Langman JS (2000). ABC of colorectal cancer: epidemiology. BMJ.

[2] Haggar FA, Boushey RP (2009). Colorectal cancer epidemiology: incidence, mortality, survival, and risk factors. Clin Colon Rectal Surg.

[3] Calvert PM, Frucht H (2002). The genetics of colorectal cancer. Ann Intern Med.

[4] Morin PJ, Sparks AB, Korinek V, Barker N, Clevers H, Vogelstein B, Kinzler KW (1997). Activation of beta-catenin-Tcf signaling in colon cancer by mutations in beta-catenin or APC. Science (New York, NY).

[5] Jinushi M, Baghdadi M, Chiba S, Yoshiyama H (2012). Regulation of cancer stem cell activities by tumor-associated macrophages. Am J Cancer Res.

[6] Sylvester KG, Colnot S (2014). Hippo/YAP, beta-catenin, and the cancer cell: a “menage a trois” in hepatoblastoma. Gastroenterology.

[7] Tao J, Calvisi DF, Ranganathan S, Cigliano A, Zhou L, Singh S, Jiang L, Fan B, Terracciano L, Armeanu-Ebinger S (2014). Activation of beta-catenin and Yap1 in human hepatoblastoma and induction of hepatocarcinogenesis in mice. Gastroenterology.

[8] Nussinov R, Tsai CJ, Jang H, Korcsmaros T, Csermely P (2016). Oncogenic KRAS signaling and YAP1/beta-catenin: similar cell cycle control in tumor initiation. Semin Cell Dev Biol..

[9] Cai J, Maitra A, Anders RA, Taketo MM, Pan D (2015). Beta-catenin destruction complex-independent regulation of Hippo-YAP signaling by APC in intestinal tumorigenesis. Genes Dev.

[10] Bamodu OA, Huang WC, Tzeng DT, Wu A, Wang LS, Yeh CT, Chao TY (2015). Ovatodiolide sensitizes aggressive breast cancer cells to doxorubicin, eliminates their cancer stem cell-like phenotype, and reduces doxorubicin-associated toxicity. Cancer Lett.

[11] Liao YF, Rao YK, Tzeng YM (2012). Aqueous extract of Anisomeles indica and its purified compound exerts anti-metastatic activity through inhibition of NF-kappaB/AP-1-dependent MMP-9 activation in human breast cancer MCF-7 cells. Food Chem Toxicol.

[12] Ho JY, Hsu RJ, Wu CL, Chang WL, Cha TL, Yu DS, Yu CP (2013). Ovatodiolide targets beta-catenin signaling in suppressing tumorigenesis and overcoming drug resistance in renal cell carcinoma. Evid Based Complement Alternat Med.

[13] Hsieh YJ, Tseng SP, Kuo YH, Cheng TL, Chiang CY, Tzeng YM, Tsai WC (2016). Ovatodiolide of Anisomeles indica exerts the anticancer potential on pancreatic cancer cell lines through STAT3 and NF-kappaB regulation. Evid Based Complement Alternat Med.

[14] Lu KT, Wang BY, Chi WY, Chang-Chien J, Yang JJ, Lee HT, Tzeng YM, Chang WW (2016). Ovatodiolide inhibits breast cancer stem/progenitor cells through SMURF2-mediated downregulation of Hsp27. Toxins.

[15] Lin KL, Tsai PC, Hsieh CY, Chang LS, Lin SR (2011). Antimetastatic effect and mechanism of ovatodiolide in MDA-MB-231 human breast cancer cells. Chem Biol Interact.

[16] Wu T, Dai Y, Wang W, Teng G, Jiao H, Shuai X, Zhang R, Zhao P, Qiao L (2016). Macrophage targeting contributes to the inhibitory effects of embelin on colitis-associated cancer. Oncotarget.

[17] Kaiser S, Park YK, Franklin JL, Halberg RB, Yu M, Jessen WJ, Freudenberg J, Chen X, Haigis K, Jegga AG (2007). Transcriptional recapitulation and subversion of embryonic colon development by mouse colon tumor models and human colon cancer. Genome Biol.

[18] Loboda A, Nebozhyn MV, Watters JW, Buser CA, Shaw PM, Huang PS, Van’t Veer L, Tollenaar RA, Jackson DB, Agrawal D (2011). EMT is the dominant program in human colon cancer. BMC Med Genomics.

[19] Kim T, Yang SJ, Hwang D, Song J, Kim M, Kyum Kim S, Kang K, Ahn J, Lee D, Kim MY (2015). A basal-like breast cancer-specific role for SRF-IL6 in YAP-induced cancer stemness. Nat Commun.

[20] Lee KW, Lee SS, Kim SB, Sohn BH, Lee HS, Jang HJ, Park YY, Kopetz S, Kim SS, Oh SC (2015). Significant association of oncogene YAP1 with poor prognosis and cetuximab resistance in colorectal cancer patients. Clin Cancer Res.

[21] Xu H, Lai W, Zhang Y, Liu L, Luo X, Zeng Y, Wu H, Lan Q, Chu Z (2014). Tumor-associated macrophage-derived IL-6 and IL-8 enhance invasive activity of LoVo cells induced by PRL-3 in a KCNN4 channel-dependent manner. BMC Cancer.

[22] Ho JY, Hsu RJ, Wu CL, Chang WL, Cha TL, Yu DS, Yu CP (2013). Ovatodiolide targets beta-catenin signaling in suppressing tumorigenesis and overcoming drug resistance in renal cell carcinoma. Evid Based Complement Alternat Med.

[23] Huang HC, Lien HM, Ke HJ, Chang LL, Chen CC, Chang TM (2012). Antioxidative characteristics of Anisomeles indica extract and inhibitory effect of ovatodiolide on melanogenesis. Int J Mol Sci.

[24] Zhou Q, Peng RQ, Wu XJ, Xia Q, Hou JH, Ding Y, Zhou QM, Zhang X, Pang ZZ, Wan DS (2010). The density of macrophages in the invasive front is inversely correlated to liver metastasis in colon cancer. J Transl Med.

[25] O’Hagan-Wong K, Nadeau S, Carrier-Leclerc A, Apablaza F, Hamdy R, Shum-Tim D, Rodier F, Colmegna I (2016). Increased IL-6 secretion by aged human mesenchymal stromal cells disrupts hematopoietic stem and progenitor cells’ homeostasis. Oncotarget.

[26] Waldner MJ, Foersch S, Neurath MF (2012). Interleukin-6—a key regulator of colorectal cancer development. Int J Biol Sci.

[27] Sica A, Rubino L, Mancino A, Larghi P, Porta C, Rimoldi M, Solinas G, Locati M, Allavena P, Mantovani A (2007). Targeting tumour-associated macrophages. Expert Opin Ther Targets.

[28] Siveen KS, Kuttan G (2009). Role of macrophages in tumour progression. Immunol Lett.

[29] Edin S, Wikberg ML, Dahlin AM, Rutegard J, Oberg A, Oldenborg PA, Palmqvist R (2012). The distribution of macrophages with a M1 or M2 phenotype in relation to prognosis and the molecular characteristics of colorectal cancer. PLoS One.

[30] Zhu P, Baek SH, Bourk EM, Ohgi KA, Garcia-Bassets I, Sanjo H, Akira S, Kotol PF, Glass CK, Rosenfeld MG (2006). Macrophage/cancer cell interactions mediate hormone resistance by a nuclear receptor derepression pathway. Cell.

[31] Biswas SK, Allavena P, Mantovani A (2013). Tumor-associated macrophages: functional diversity, clinical significance, and open questions. Semin Immunopathol.

[32] Nagasaki T, Hara M, Nakanishi H, Takahashi H, Sato M, Takeyama H (2014). Interleukin-6 released by colon cancer-associated fibroblasts is critical for tumour angiogenesis: anti-interleukin-6 receptor antibody suppressed angiogenesis and inhibited tumour-stroma interaction. Br J Cancer.

[33] Bingle L, Brown NJ, Lewis CE (2002). The role of tumour-associated macrophages in tumour progression: implications for new anticancer therapies. J Pathol.

[34] Cieslewicz M, Tang J, Yu JL, Cao H, Zavaljevski M, Motoyama K, Lieber A, Raines EW, Pun SH (2013). Targeted delivery of proapoptotic peptides to tumor-associated macrophages improves survival. Proc Natl Acad Sci U S A.

[35] Rao G, Wang H, Li B, Huang L, Xue D, Wang X, Jin H, Wang J, Zhu Y, Lu Y (2013). Reciprocal interactions between tumor-associated macrophages and CD44-positive cancer cells via osteopontin/CD44 promote tumorigenicity in colorectal cancer. Clin Cancer Res.

[36] Sica A, Mantovani A (2012). Macrophage plasticity and polarization: in vivo veritas. J Clin Invest.

[37] Tang X, Mo C, Wang Y, Wei D, Xiao H (2013). Anti-tumour strategies aiming to target tumour-associated macrophages. Immunology.

[38] Covarrubias AJ, Aksoylar HI, Yu J, Snyder NW, Worth AJ, Iyer SS, Wang J, Ben-Sahra I, Byles V, Polynne-Stapornkul T (2016). Akt-mTORC1 signaling regulates Acly to integrate metabolic input to control of macrophage activation. elife.

[39] Barrett JP, Minogue AM, Falvey A, Lynch MA (2015). Involvement of IGF-1 and Akt in M1/M2 activation state in bone marrow-derived macrophages. Exp Cell Res.

[40] Byles V, Covarrubias AJ, Ben-Sahra I, Lamming DW, Sabatini DM, Manning BD, Horng T (2013). The TSC-mTOR pathway regulates macrophage polarization. Nat Commun.

[41] Shao DD, Xue W, Krall EB, Bhutkar A, Piccioni F, Wang X, Schinzel AC, Sood S, Rosenbluh J, Kim JW (2014). KRAS and YAP1 converge to regulate EMT and tumor survival. Cell.

[42] Di Agostino S, Sorrentino G, Ingallina E, Valenti F, Ferraiuolo M, Bicciato S, Piazza S, Strano S, Del Sal G, Blandino G (2016). YAP enhances the pro-proliferative transcriptional activity of mutant p53 proteins. EMBO Rep.

[43] Dotse E, Bian Y (2016). Isolation of colorectal cancer stem-like cells. Cytotechnology.

[44] Rao YK, Fang SH, Hsieh SC, Yeh TH, Tzeng YM (2009). The constituents of Anisomeles indica and their anti-inflammatory activities. J Ethnopharmacol.

[45] Vichai V, Kirtikara K (2006). Sulforhodamine B colorimetric assay for cytotoxicity screening. Nat Protoc.

[46] Tjiu JW, Chen JS, Shun CT, Lin SJ, Liao YH, Chu CY, Tsai TF, Chiu HC, Dai YS, Inoue H (2009). Tumor-associated macrophage-induced invasion and angiogenesis of human basal cell carcinoma cells by cyclooxygenase-2 induction. J Invest Dermatol.

[47] Yeh CT, Wu AT, Chang PM, Chen KY, Yang CN, Yang SC, Ho CC, Chen CC, Kuo YL, Lee PY (2012). Trifluoperazine, an antipsychotic agent, inhibits cancer stem cell growth and overcomes drug resistance of lung cancer. Am J Respir Crit Care Med.

[48] Franken NA, Rodermond HM, Stap J, Haveman J, van Bree C (2006). Clonogenic assay of cells in vitro. Nat Protoc.

[49] McDonald JW, Pilgram TK (1999). Nuclear expression of p53, p21 and cyclin D1 is increased in bronchioloalveolar carcinoma. Histopathology.

