# Supplementary material for: Ovatodiolide suppresses colon tumorigenesis and prevents polarization of M2 tumor-associated macrophages through YAP oncogenic pathways
Source: J Hematol Oncol. 2017 Feb 28;10:60. doi: 10.1186/s13045-017-0421-3 (PMC5329923; doi:10.1186/s13045-017-0421-3)
Supplement: Additional file 1: Figure S2. — YAP1 expression is associated with M1-M2 polarization. (A) YAP1 silencing in macrophages (M0) leads to decreased M2 polarization. Real-time PCR analysis demonstrates that M2 markers, TGF-b1 and Ym2, were significantly reduced while one of the M1 marker iNOS was significantly increased, in the macrophages generated in the presence of DLD-1 cells. (B) YAP1-overexpressing colon cancer cells also promote the polarization of M2 genotype in THP-1 cells. Experiments were performed at least three times. *P ≤ 0.05, **P ≤ 0.01, ***P ≤ 0.001. The primer sequences used in the q-PCR experiments are shown. (PPTX 109 kb) [file 13045_2017_421_MOESM1_ESM.pptx]

## Slide 1
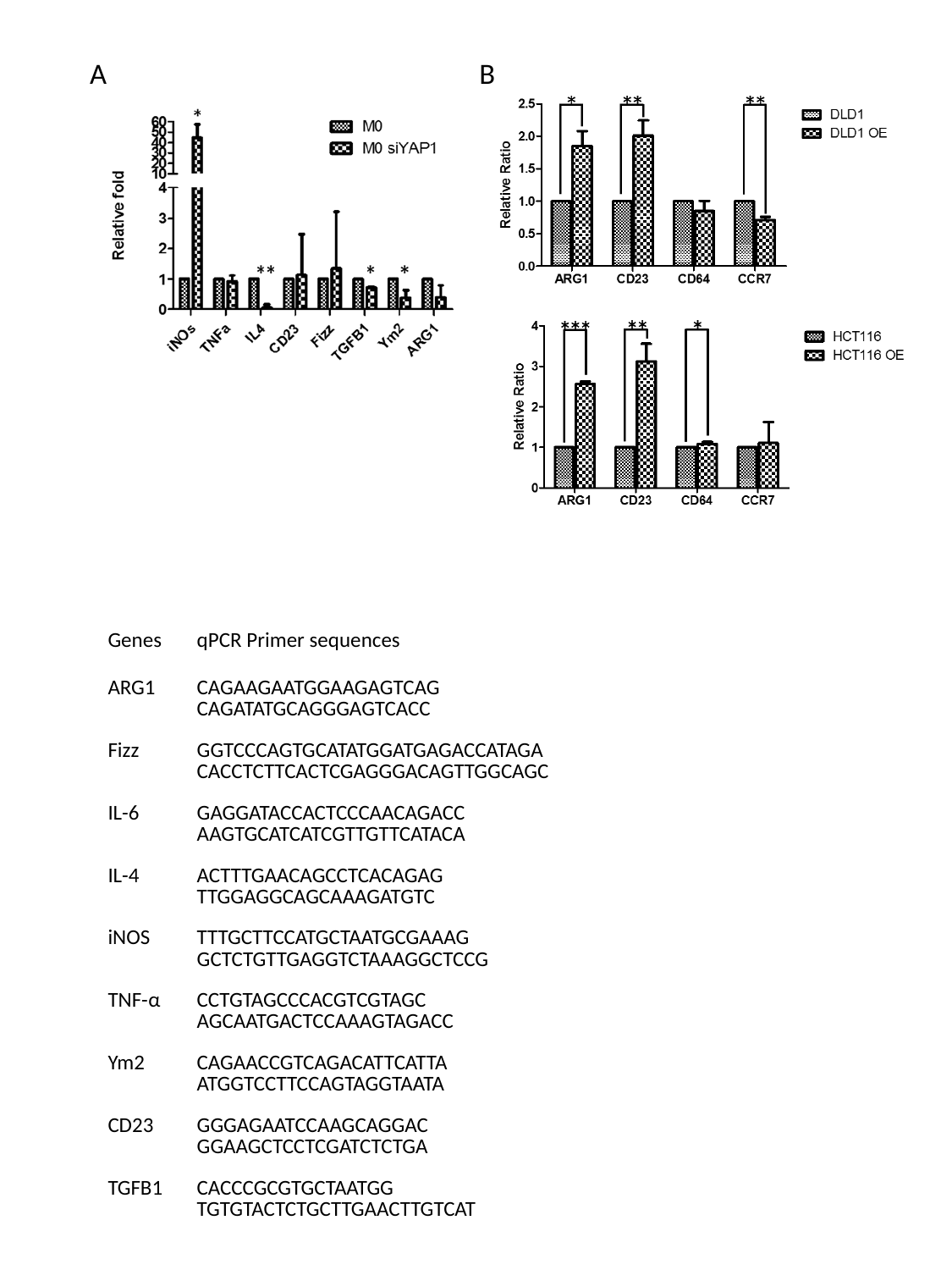

A
B
| Genes | qPCR Primer sequences |
| --- | --- |
| ARG1 | CAGAAGAATGGAAGAGTCAG CAGATATGCAGGGAGTCACC |
| Fizz | GGTCCCAGTGCATATGGATGAGACCATAGA CACCTCTTCACTCGAGGGACAGTTGGCAGC |
| IL-6 | GAGGATACCACTCCCAACAGACC AAGTGCATCATCGTTGTTCATACA |
| IL-4 | ACTTTGAACAGCCTCACAGAG TTGGAGGCAGCAAAGATGTC |
| iNOS | TTTGCTTCCATGCTAATGCGAAAG GCTCTGTTGAGGTCTAAAGGCTCCG |
| TNF-α | CCTGTAGCCCACGTCGTAGC AGCAATGACTCCAAAGTAGACC |
| Ym2 | CAGAACCGTCAGACATTCATTA ATGGTCCTTCCAGTAGGTAATA |
| CD23 | GGGAGAATCCAAGCAGGAC GGAAGCTCCTCGATCTCTGA |
| TGFB1 | CACCCGCGTGCTAATGG TGTGTACTCTGCTTGAACTTGTCAT |
