# Supplementary figures and images for: Ovatodiolide suppresses colon tumorigenesis and prevents polarization of M2 tumor-associated macrophages through YAP oncogenic pathways
Source: J Hematol Oncol. 2017 Feb 28;10:60. doi: 10.1186/s13045-017-0421-3 (PMC5329923; doi:10.1186/s13045-017-0421-3)

## Slide 1
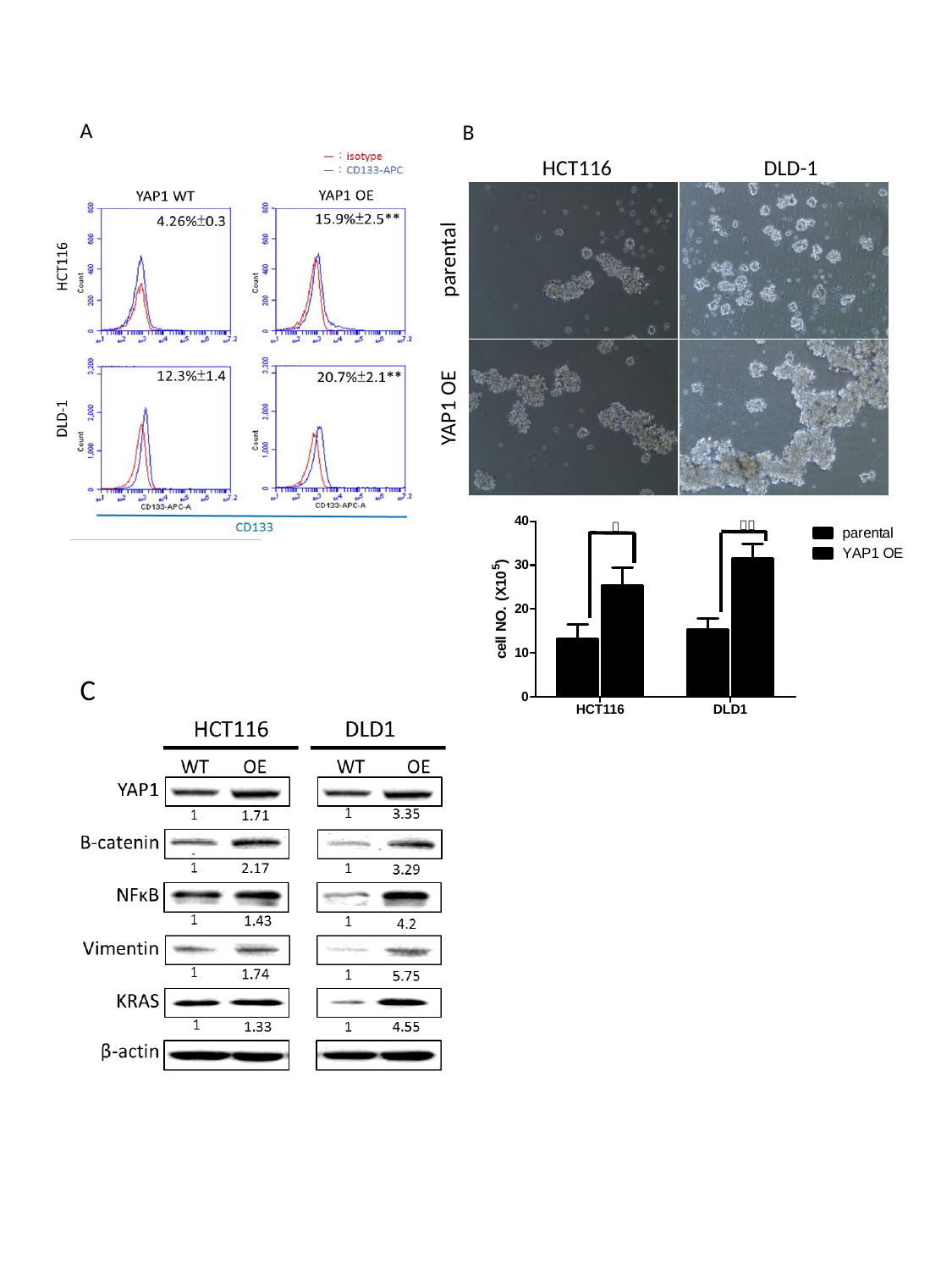

A
B
HCT116
DLD-1
parental
YAP1 OE


C

Supplement: Additional file 2: Figure S1. — YAP1 overexpression (OE) leads to increased stemness in colon cancer cells. (A) A significantly increased CD133+ cell percentage was found in the YAP1 OE cells as compared to their wild-type counterparts as demonstrated by our flow cytometric analysis. (B) YAP1-overexpressing HCT116 and DLD-1 cells were found to form a significantly higher number of tumor spheres (*P ≤ 0.05, **P ≤ 0.01). (C) Comparative western blots of wild-type and YAP1 overexpressing HCT116 and DLD-1 cells. An increased expression of β-catenin, NF-kB, vimentin, and Kras is associated with YAP1 overexpression. (PPTX 396 kb) [file 13045_2017_421_MOESM2_ESM.pptx]
